# Risk factors for mortality among hospitalized COVID-19 patients in Northern Ethiopia: A retrospective analysis

**DOI:** 10.1371/journal.pone.0271124

**Published:** 2022-08-11

**Authors:** Haftom Temesgen Abebe, Afework Mulugeta, Yibrah Berhe, Kiros Berhane, Amir Siraj, Dawd Siraj, Maru Aregawi, Berhane Fseha, Mohamedawel Mohamedniguss Ebrahim, Solomon Hintsa, Hagazi Gebre, Abrahim Hassen Mohammed, Hagos Godefay

**Affiliations:** 1 School of Public Health, College of Health Sciences, Mekelle University, Mekelle, Ethiopia; 2 Laboratory Interdisciplinary Statistical Data Analysis, College of Health Sciences, Mekelle University, Mekelle, Ethiopia; 3 School of Medicine, College of Health Sciences, Mekelle University, Mekelle, Ethiopia; 4 Department of Biostatistics, Mailman School of Public Health, Columbia University, New York, NY, United States of America; 5 Department of Biological Sciences and Notre Dame Environmental Change Initiative, University of Notre Dame, Notre Dame, IN, United States of America; 6 Division of Infectious Diseases, University of Wisconsin-Madison, Madison, Wisconsin, United States of America; 7 Global Malaria Program, World Health Organization, Geneva, Switzerland; 8 Department of Public Health, College of Health Sciences, Adigrat University, Adigrat, Ethiopia; 9 Department of Epidemiology and Biostatistics, School of Public Health, College of Health Sciences, Aksum University, Aksum, Ethiopia; 10 Tigray Health Bureau, Mekelle, Tigray, Ethiopia; Aga Khan University - Kenya, KENYA

## Abstract

**Background:**

COVID-19 is a deadly pandemic caused by an RNA virus that belongs to the family of CORONA virus. To counter the COVID-19 pandemic in resource limited settings, it is essential to identify the risk factors of COVID-19 mortality. This study was conducted to identify the social and clinical determinants of mortality in COVID-19 patients hospitalized in four treatment centers of Tigray, Northern Ethiopia.

**Methods:**

We reviewed data from 6,637 COVID-19 positive cases that were reported from May 7, 2020 to October 28, 2020. Among these, 925 were admitted to the treatment centers because of their severity and retrospectively analyzed. The data were entered into STATA 16 version for analysis. The descriptive analysis such as median, interquartile range, frequency distribution and percentage were used. Binary logistic regression model was fitted to identify the potential risk factors of mortality of COVID-19 patients. The adjusted odds ratio (AOR) with 95% confidence interval was used to determine the magnitude of the association between the outcome and predictor variables.

**Results:**

The median age of the patients was 30 years (IQR, 25–44) and about 70% were male patients. The patients in the non-survivor group were much older than those in the survivor group (median 57.5 years versus 30 years, *p*-value < 0.001). The overall case fatality rate was 6.1% (95% CI: 4.5% - 7.6%) and was increased to 40.3% (95% CI: 32.2% - 48.4%) among patients with critical and severe illness. The proportions of severe and critical illness in the non-survivor group were significantly higher than those in the survivor group (19.6% versus 5.1% for severe illness and 80.4% versus 4.5% for critical illness, all *p*-value < 0.001). One or more pre-existing comorbidities were present in 12.5% of the patients: cardiovascular diseases (42.2%), diabetes mellitus (25.0%) and respiratory diseases (16.4%) being the most common comorbidities. The comorbidity rate in the non-survivor group (44.6%) was higher than in the survivor group (10.5%). The results from the multivariable binary regression showed that the odds of mortality was higher for patients who had cardiovascular diseases (AOR = 2.49, 95% CI: 1.03–6.03), shortness of breath (AOR = 9.71, 95% CI: 4.73–19.93) and body weakness (AOR = 3.04, 95% CI: 1.50–6.18). Moreover, the estimated odds of mortality significantly increased with patient’s age.

**Conclusions:**

Age, cardiovascular diseases, shortness of breath and body weakness were the predictors for mortality of COVID-19 patients. Knowledge of these could lead to better identification of high risk COVID-19 patients and thus allow prioritization to prevent mortality.

## Introduction

The first case of the coronavirus disease (COVID-19) was confirmed in December 2019 in Wuhan, Hubei province, China [1–3]. It evolved from wildlife [4], and can cause fever and severe respiratory syndrome in human beings and was declared as a pandemic by the World Health Organization. COVID-19 is an emerging infectious disease due to severe acute respiratory syndrome coronavirus 2 (SARS-CoV-2). It is associated with lower or upper respiratory infections [5,6]. The infection fatality rates of COVID-19 patients, patient outcomes and related complications reported so far have varied considerably between countries.

Previous studies showed that the overall case fatality rate of COVID-19 patients is 3.77% -5.4%, and 41.1% - 61.5% among critically and severely ill patients respectively [7–12]. To reduce the infection fatality rate, identifying the determinants related to mortality in COVID-19 patients is urgently needed. This is crucial for the decision-making process at national and international levels in order to properly respond to the pandemic. Although previous studies reported that old age, and underlying comorbidities were closely associated with disease severity or death of COVID-19 patients [8,11,13,14], the risk factors related to the mortality of COVID-19 patients in low and middle income countries are not well studied. Besides, the prevalence of underlying chronic non communicable diseases, known to be important risk factors for mortality in COVID-19 patients are also different between countries [15,16].

Identifying the determinants related to mortality in COVID-19 patients is urgently needed to reduce the case fatality rate of the deadly disease. The present study analyzed the social and clinical determinants of mortality among hospitalized COVID-19 patients in four treatment centers in Tigray, Ethiopia. This study provided useful information to associate risk factors with case fatality of COVID-19 hospitalized patients and support decision making regarding COVID-19 in resource limited settings.

## Materials and methods

### Study design and settings

A retrospective cohort study was used that involved all COVID-19 patients from 7 May to 28 October 2020, from six COVID-19 isolation and treatment centers namely Mekelle, Maichew, Axum, Adigrat, Shire and Humera. Maichew and Shire were isolation centers whereas Mekelle, Axum, Adigrat and Humera had both isolation and treatment centers. Patients who were in an isolation center were transferred to one of the 4 isolation and treatment centers if hospitalization needed. Following the declaration of a pandemic situation by WHO, the Tigray regional state government with Tigray Health Bureau (THB) implemented mass screening of all travelers who enter to the region, individuals who had been in contact with confirmed cases of COVID-19 and individuals in high risk settings (health care workers, private business employees, long track drivers and merchants). Regardless of sign or symptoms development, all individuals with laboratory confirmed COVID-19 infection were admitted to the isolation and treatment centers within 24 hours. Moreover, anyone who has contact with confirmed COVID-19 cases was being isolated for 14 days. Persons who failed to develop symptoms within 14 days were being discharged from the isolation centers. Cases were confirmed by polymerase chain reaction (PCR) in the treatment centers.

### Study participants and study period

The study participants were all laboratory-confirmed positive cases of SARS-CoV-2 admitted to the six isolation and treatment centers between May 7 and October 28, 2020.

### Data source and sample

The data were collected using standardized form from electronic medical records from the six isolation and treatment centers. The data set contains clinical information of the patients, demographic characteristics and patient outcomes. All laboratory-confirmed COVID-19 cases admitted to the isolation and treatment centers and who were candidates for hospitalization were included in this study.

### Operational definitions

COVID-19 cases are all individuals tested positive for SARS-CoV-2 by polymerase chain reaction (PCR) in the isolation and treatment centers. Symptomatic case is defined as any SARS-CoV-2 positive individual by PCR in the treatment centers with at least one sign or symptom for COVID-19. Signs and symptoms of COVID-19 include but not limited to: fever, cough, shortness of breath, headache, sore throat, pain, fatigue, myalgia, nasal congestion, diarrhea, nausea, vomiting, loss of smell, loss of taste and loss of appetite. Severe cases are with clinical signs of pneumonia (fever, cough, dyspnea, fast breathing) and have one of the following conditions: i) respiratory rate interval > 30 breaths/min; ii) SpO2 (saturation of peripheral oxygen) < 93% at rest; iii) severe respiratory distress. Critical cases have to meet one of the following conditions: i) respiratory failure and consequent needs of mechanical ventilation; ii) shock; iii) require intensive care because of multiple organ dysfunction. Asymptomatic patient is any patient who tested positive for COVID-19 but does not have any of the symptoms. These patients are detected after isolation and contact tracing. Cases with comorbidity are COVID-19 patients with at least one known preexisting chronic medical illness.

### Study variables

#### Dependent variable

The dependent variable in this study was hospitalized COVID-19 patient outcome. It was dichotomized as 1 if the patient has died and 0 if the patient has recovered. The confirmed COVID-19 patients after spending some days in isolation and treatment centers were retested and they can be discharged when the symptoms have subsided, the body temperature remains at a normal range for at least three days, two consecutive laboratory tests are negative and radiological improvement.

#### Independent variables

In this study the independent variables were sex, age, occupation, signs and symptoms, comorbidity and type of comorbidity, disease severity status, temperature, travel history, nationality and source of infection.

### Statistical data analysis

The data were coded, cleaned and checked for completeness. STATA version 16 software was used for data processing and data analysis. Continuous variables were presented as median and interquartile range (IQR), while categorical variables were described as frequencies (%) and compared using the chi-square test or Fisher’s exact test. Binary logistic regression model was used to explore the risk factors of mortality of COVID-19 patients. As the maximum likelihood estimation (MLE) method are systematically biased for rare events, the penalized maximum likelihood estimation (PMLE) method was used to estimate logistic regression model [17]. The PMLEs gives unbiased estimates for rare events and always converged even for small sample size. Bivariate analysis was first used to identify independent variables that were associated with the outcome i.e. death versus recovered. Independent variables that were significant at 0.25 levels in the bivariate analysis were further included in to multivariable binary logistic regression. Odds ratio with 95% confidence interval was also used to measure the degree of association between predictors and outcome variable, and the assumptions of multicollinearity between two or more independent variables were checked using variance inflation factors (VIFs). Goodness of fit of the model was assessed using Hosmer-Lemeshow test. The discriminatory accuracy of a diagnostic test was also assessed through receiver operating curve (ROC).

### Ethical considerations

Permission to assess the data was obtained from Tigray Health Bureau (THB) and Mekelle University. This study was approved by the research ethics review committee of the College of Health Sciences, Mekelle University with reference number: IRB 1826/2021. The Tigray Health Bureau waived the requirement informed consent before the study started due to the urgent need to collect epidemiological and clinical data. The confidentiality of data was kept as there were no personal identifiers used and neither the raw data nor the extracted data were passed to a third person.

## Results

### Socio-demographic characteristics

**Fig 1:**
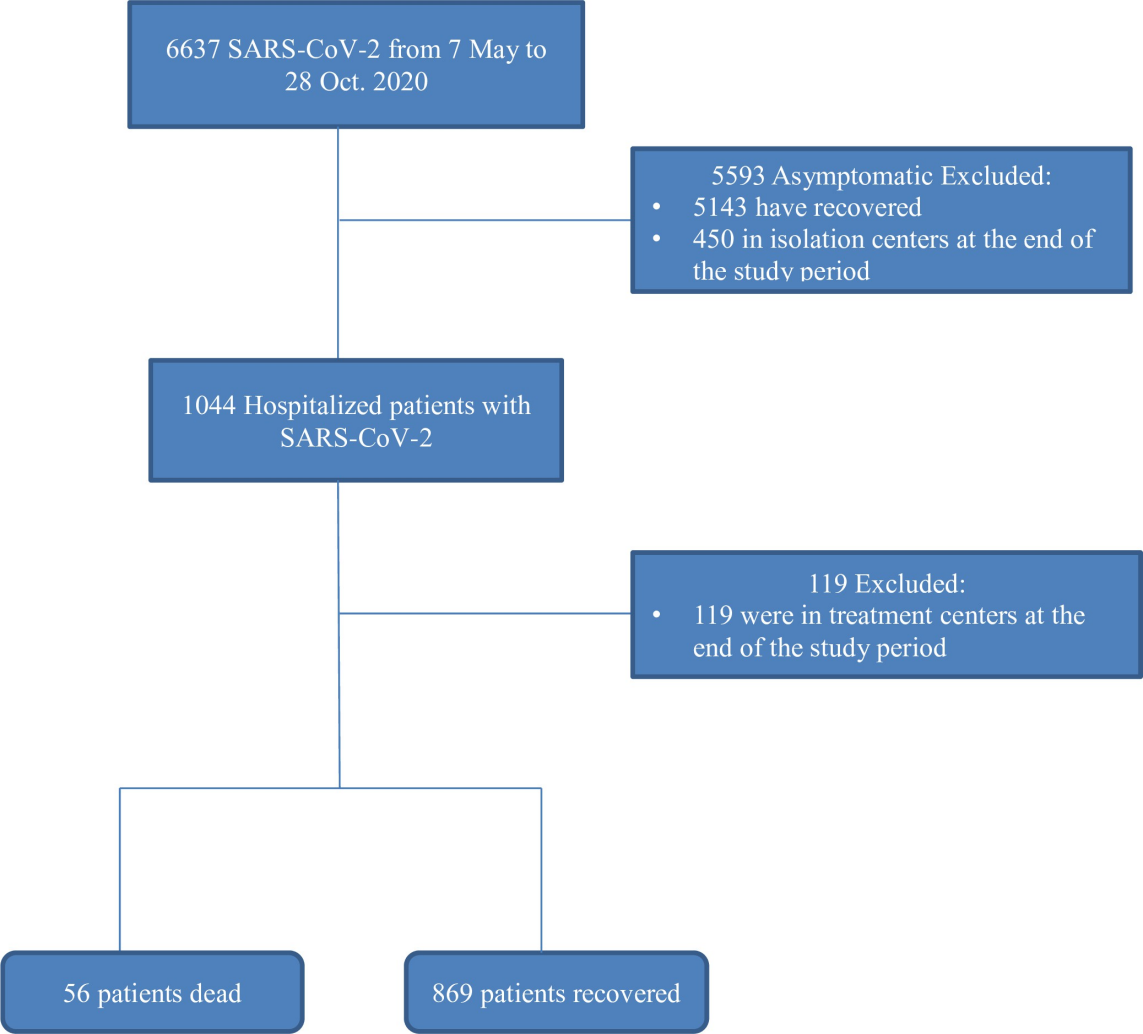
Flowchart of the study design and participants. Only the rounded rectangles were included in the analysis.

During the study period, 6,637 COVID-19 positive cases were reported from the six isolation and treatment centers, of which 1,044 were symptomatic and needed hospitalization. Of these 119 were still on treatment at the end of the study period. In this study 925 hospitalized patients who had a definite outcome (dead or recovered) were included (Fig 1). The median age of the patients was 30 years (IQR, 25–44) and 70.1% (95% CI: 67.1%-73.0%) were male patients. The patients in the non-survivor group were much older than those in the survivor group [median = 57.5 years (IQR, 29.5–72 years) versus median = 30 years (IQR, 24–42 years), *p*-value < 0.001]. A total of 45.6% (95% CI: 42.4% - 48.8%) patients were younger than 30 years, 35.2% (95% CI: 32.2% - 38.3%) were aged between 30 and 49 years, 13.6% (95% CI: 11.4% - 15.8%) were between 50 and 69 years and 5.5% (95% CI: 4.0% - 7.0%) were older than 69 years. About 10% of the patients had travel history and 10.5% were health care workers (Table 1).

**Table 1:** Socio-demographic characteristics of 925 hospitalized COVID-19 patients of Tigray, Northern Ethiopia, 2020.

Variable		Total (%)	Outcome		*P*-value
			Non-survivors (n = 56)	Survivors (n = 869)	
**Sex**					0.594
	Female	277(29.95%)	15(26.79%)	262(30.15%)	
	Male	648(70.05%)	41(73.21%)	607(69.85%)	
**Age, years**					<0.001
	<30	422(45.62%)	14(25.00%)	408(46.95%)	
	30–49	326(35.24%)	11(19.64%)	315(36.25%)	
	50–69	126(13.62%)	12(21.43%)	114(13.12%)	
	70+	51(5.51%)	19(33.93%)	32(3.68%)	
**Nationality**					0.161*
	Ethiopian	913(98.70%)	54(96.43%)	859(98.85%)	
	Foreign	12(1.30%)	2(3.57%)	10(1.15%)	
**Occupation**					0.081
	Health care workers	97(10.49%)	2(3.57%)	95(10.93%)	
	Non-health care workers	828(89.51%)	54(96.43%)	774(89.07%)	
**Travel history**					0.106
	No	834(90.16%)	47(83.93%)	787(90.56%)	
	Yes	91(9.84%)	9(16.07%)	82(9.44%)	

*: Fisher exact *p*-value.

### Clinical characteristics

**Fig 2:**
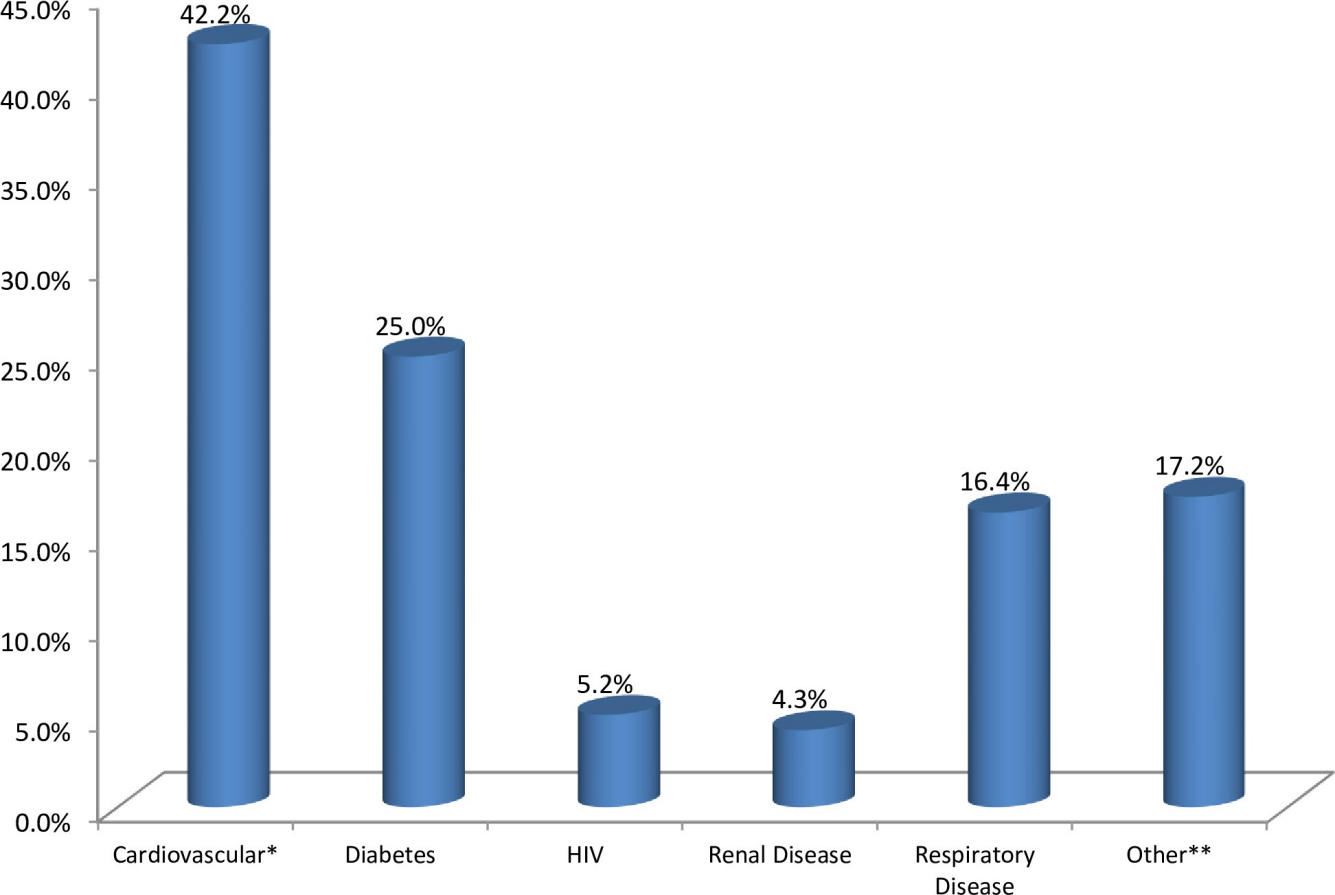
Types of underline comorbidity COVID-19 patients inTigray, Ethiopia, 2020. * Cardiovascular disease included hypertension and heart failure. ** Other: Traumatic injury, gastritis, pregnancy, allergic, psytotic disorder, rabies, arthritis, epilepsy, neurological.

The source of infection for the majority (68.4%) of the hospitalized COVID-19 patients was the community (Table 2). From the total, 56 (6.1%) patients have died. The most frequently reported signs and symptoms on admission were: cough (70.6%), fever (40.1%), sore throat (26.9%) and body weakness (25.9%). Based on the body temperature on admission, 403 (73.8%) of the patients had temperature < 37.3°C, 77 (14.1%) had temperature > 38 ^0^C. One or more pre-existing comorbidities were present in 116 (12.5%) patients: cardiovascular diseases (42.2%), diabetes mellitus (25.0%) and respiratory disease (16.4%) being the most common (Fig 2). The comorbidity rate in the non-survivor group was higher than in the survivor group (44.6% versus 10.5%, *p*-value < 0.001). Moreover, the disease severity status between groups, the proportions of severely and critically ill patients in the non-survivor group were significantly higher than those in the survivor group (19.6% versus 5.1% for severe illness and 80.4% versus 4.5% for critical illness, all *p*-value < 0.001). The case fatality rate among patients with severe illness and critically ill were 20.0% and 53.6%, respectively. The overall case fatality rate was 6.1% and was increased to 40.3% among patients with critical and severe illness (Table 2).

**Table 2:** Clinical characteristics of 925 hospitalized COVID-19 patients of Tigray, Northern Ethiopia, 2020.

Variable		Total (%)	Outcome		*P*-value
			Non-survivor (n = 56)	Survivor (n = 869)	
**Fever**					0.879
	No	554(59.89%)	33(58.93%)	521(59.95%)	
	Yes	371(40.11%)	23(41.07%)	348(40.05%)	
**Cough**					0.888
	No	272(29.41%)	16(28.57%)	256(29.46%)	
	Yes	653(70.59%)	40(71.43%)	613(70.54%)	
**Shortness of breath**					<0.001
	No	729(78.81%)	12(21.43%)	717(82.51%)	
	Yes	196(21.19%)	44(78.57%)	152(17.49%)	
**Sore throat**					0.774
	No	676(73.08%)	40(71.43%)	636(73.19%)	
	Yes	249(26.92%)	16(28.57%)	233(26.81%)	
**Headache**					0.021
	No	752(81.30%)	39(69.64%)	713(82.05%)	
	Yes	173(18.70%)	17(30.36%)	156(17.95%)	
**Body weakness**					<0.001
	No	685(74.05%)	26(46.43%)	659(75.83%)	
	Yes	240(25.95%)	30(53.57%)	210(24.17%)	
**Pain**					0.026
	No	720(78.01%)	37(66.07%)	683(78.78%)	
	Yes	203(21.99%)	19(33.93%)	184(21.22%)	
**Other symptoms** ***					1.000*
	No	904(97.73%)	55(98.21%)	849(97.70%)	
	Yes	21(2.27%)	1(1.79%)	20(2.30%)	
**Comorbidity**					<0.001
	No	809(87.46%)	31(55.36%)	778(89.53%)	
	Yes	116(12.54%)	25(44.64%)	91(10.47%)	
**Covid-19 severity**					<0.001*
	Mild	783(84.65%)	0(0.0%)	783(90.10%)	
	Moderate	3(0.32%)	0(0.0%)	3(0.35%)	
	Severe	55(5.95%)	11(19.64%)	44(5.06%)	
	Critical	84(9.08%)	45(80.36%)	39(4.49%)	
**Temperature, °C** **					0.186*
	<37.3	403(73.81%)	29(85.29%)	374(73.05%)	
	37.3–38	66(12.09%)	2(5.88%)	64(12.50%)	
	38.1–39	71(13.00%)	2(5.88%)	69(13.48%)	
	> 39	6(1.10%)	1(2.94%)	5(0.98%)	
**Source of infection**					0.120
	Community	633(68.43%)	42(75.00%)	591(68.01%)	
	Contact	195(21.08%)	6(10.71%)	189(21.75%)	
	Imported	97(10.49%)	8(14.29%)	89(10.24%)	

*: Fisher exact *p*-value

**: 41% missing values existed in this variable

*** Other symptoms: Vomiting, chills, loss of smell, loss of taste, diarrhea, loss of appetite, abdominal cramping, myalgia.

### Risk factors of mortality among hospitalized COVID-19 patients

To determine the potential risk factors of mortality among the hospitalized COVID-19 patients, binary logistic regression model was conducted. We initially performed bivariate analysis followed by multivariable analysis. Predictors that were statistically associated with the outcome at *p*-value < 0.25 in the bivariate analysis were further included to the multivariable analysis. The multivariable binary logistic regression analysis showed that age, shortness of breath, body weakness and cardiovascular diseases were found as significant predictors of mortality of COVID-19 patients (Table 3). Keeping the effect of other predictors constant, COVID-19 patients with age < 30 years had 79% times (AOR = 0.21, 95% CI: 0.08–0.53) lower odds to die and patients with age 30–49 years were 0.14 times (AOR = 0.14, 95% CI: 0.06–0.36) less likely to die compared to patients with age 70 and above years. In addition, COVID-19 patients who had cardiovascular disease were 2.7 times (AOR = 2.49, 95% CI: 1.03–6.03) more likely to die than patients who had no cardiovascular disease. Patients who had shortness of breath at admission were 9.7 times (AOR = 9.71, 95% CI: 4.73–19.93) more likely to die than their counterpart. COVID-19 patients who had body weakness at admission were 3 times (AOR = 3.04, 95% CI: 1.50–6.18) higher odds to die than those who had no body weakness. The receiver operating curve (ROC) analysis showed a very good accuracy in classifying mortality due to COVID-19 (area under the curve = 0.8713). Finally, Hosmer and Lemeshow test results confirmed that the model was a good fit for the data (*X*^2^(9) = 7.82, *p*-value = 0.553).

**Table 3:** Multivariable binary logistic analysis of factors associated with mortality among hospitalized COVID-19 patients, Tigray, Northern Ethiopia, 2020.

Variable		Total (%)	COR (95% CI)	*P*-value	AOR (95% CI)	*P*-value	VIF
**Sex**							
	Female (ref.)	277(29.95%)	1(ref)				
	Male	648(70.05%)	1.16(0.63–2.11)	0.635			
**Age, years**							1.19
	< 30	422(45.62%)	0.06(0.03–0.13)	< 0.001	0.21(0.08–0.53)	0.001	
	30–49	326(35.24%)	0.06(0.03–0.14)	< 0.001	0.14(0.06–0.36)	< 0.001	
	50–69	126(13.62%)	0.18(0.08–0.41)	< 0.001	0.27(0.11–0.69)	0.006	
	70+ (ref.)	51(5.51%)	1(ref)		1(ref)		
**Fever**							
	No (ref.)	554(59.89%)	1(ref)				
	Yes	371(40.11%)	1.05(0.61–1.81)	0.932			
**Cough**							
	No (ref.)	272(29.41%)	1(ref)				
	Yes	653(70.59%)	1.03(0.57–1.85)				
**Shortness of breath**							1.19
	No (ref.)	729(78.81%)	1(ref)		1(ref)		
	Yes	196(21.19%)	16.75(8.73–32.13)	< 0.001	9.71(4.73–19.93)	< 0.001	
**Sore throat**							
	No (ref.)	676(73.08%)	1(ref)				
	Yes	249(26.92%)	1.11(0.62–2.01)	0.728			
**Headache**							1.24
	No (ref.)	752(81.30%)	1(ref)		1(ref)		
	Yes	173(18.70%)	2.02(1.12–3.64)	0.019	0.51(0.22–1.18)	0.116	
**Body weakness**							1.21
	No (ref.)	685(74.05%)	1(ref)		1(ref)		
	Yes	240(25.95%)	3.61(2.10–6.21)	< 0.001	3.04(1.50–6.18)	0.002	
**Pain**							1.19
	No (ref.)	720(78.01%)	1(ref)		1(ref)		
	Yes	203(21.99%)	1.93(1.09–3.41)	0.024	1.25(0.58–2.74)	0.563	
**Cardiovascular diseases**							1.16
	No (ref.)	876(94.70%)	1(ref)		1(ref)		
	Yes	49(5.30%)	8.02(4.05–15.90)	< 0.001	2.49(1.03–6.03)	0.043	
**Diabetes mellitus**							1.04
	No (ref.)	876(94.70%)	1(ref)		1(ref)		
	Yes	49(5.30%)	8.02(4.05–15.90)	< 0.001	1.78(0.51–6.21)	0.365	
**Respiratory diseases**							1.03
	No (ref.)	896(96.86%)	1(ref)		1(ref)		
	Yes	29(3.14%)	3.67(1.40–9.69)	0.008	1.92(0.45–8.10)	0.377	
**Renal diseases**							1.02
	No (ref.)	906(97.95%)	1(ref)		1(ref)		
	Yes	19(2.05%)	4.73(1.60–14.00)	0.084	1.11(0.11–11.70)	0.933	

AOR: Adjusted odds ratio, COR: Crude odds ratio, CI: Confidence interval, ref: Reference.

## Discussion

COVID-19 is an emerging infectious disease due to severe acute respiratory syndrome coronavirus 2 (SARS-CoV-2) and has become a global pandemic. To reduce the infection fatality rate, identifying the determinants related to mortality in COVID-19 patients is urgently needed. This paper assessed the risk factors of mortality among hospitalized COVID-19 patients.

This study comprised of 925 hospitalized COVID-19 patients with 56 deaths and 869 patients discharged improved. Our study showed that men accounted for a higher proportion of COVID-19 patients than women, which was consistent with most of the confirmed cases in France, China and Italy [15,18–21]. The observed overall case fatality rate was 6.1%, which was similar with previous studies from China. The case fatality rate of COVID-19 was reported nearly 3.7–5.4 [7–9,22]. However, the magnitude was significantly lower than the previous studies conducted in France and New York City [15,23]. The discrepancy could be due to the older age of patients in these study (median ages: 72 and 63 years respectively) that could have led to high severe disease, which explain the higher mortality rates reported in these studies.

The mortality rate increased to 40.3% among patients with severe and critical illness, which was significantly higher than the study conducted in Hubei province, China [22]. However, it was lower than the study conducted in France [15]. The younger age of patients in our study (median age 30 years) could have led to lower mortality rate. Previous studies reported that non-survivor patients of COVID-19 were older [11,14,22]. Moreover, it could be due to the treatment experience and awareness during the later period of this pandemic.

In this study the case fatality rate was 53.6% among patients with critically ill patients and 20.0% among the severely ill patients. The case fatality rate among the critically ill patients was similar with previous studies [10–12,22]. Our results showed that non-survivor patients were older and they had underlying diseases. This was consistent with most previous research studies [8,11,14,15,22,24,25]. Higher proportion of cardiovascular diseases and diabetes mellitus were in non-survivor than in the survivor groups, which was similar to those reported in other studies [9,15,21,22,23–28]. As reported previously, cardiovascular diseases and diabetes mellitus were the most common comorbidities [21,26]. This is in line with our findings. Our data also showed that the most frequently reported symptoms on admission were cough, fever, sore throat, body weakness, pain and shortness of breath. As reported from previous studies the top three common symptoms were fever, cough and fatigue [22,29]. In this study there were no differences in gender, symptoms fever, cough and headache between the survivor and non-survivor groups. This finding was similar with some previous studies [13,22].

The multivariable binary logistic regression analysis demonstrated that age, shortness of breath, body weakness and cardiovascular disease were associated with the mortality of hospitalized COVID-19 patients. We found that older age was associated with high risk of mortality of COVID-19 patients, which was similar with previous studies [8,11,13,14,22]. Our study also showed that the odds of mortality were higher for patients who had cardiovascular disease, shortness of breath and body weakness at admission.

Our study is limited to the time frame of March–October 2020 because of the ongoing war in Tigray region. Since November of 2020, a destruction was launched over Tigray region by the Ethiopian and Eritrean forces with destruction of healthcare infrastructure and disruption of services [30]. COVID service has been severely affected (83%). This has impacted COVID vaccine access and vaccine coverage has remained below 2%. Future study should look at the impact of healthcare disruption on COVID-19 mortality. In addition, risk factors for COVID-19 mortality pre and post introduction of vaccine in Tigray region should be studied.

## Conclusions

In conclusion, this study showed that non-survivors hospitalized COVID-19 patients were old and they had underlying comorbidity diseases. The factors affecting mortality among COVID-19 patients were age, shortness of breath, body weakness and cardiovascular diseases. Knowledge of these could lead to better identification of high risk COVID-19 patients and thus allow prioritization to prevent mortality.

## Supporting information

S1 Dataset (DTA)

## Abbreviations

COVID-19: Coronavirus 2019
AOR: Adjusted odds ratio
CI: Confidence interval
SARS-CoV-2: severe acute respiratory syndrome corona virus 2, Tigray Health Bureau (THB)
